# Study Profile of the Japan Multi-institutional Collaborative Cohort (J-MICC) Study

**DOI:** 10.2188/jea.JE20200147

**Published:** 2021-12-05

**Authors:** Kenji Takeuchi, Mariko Naito, Sayo Kawai, Mineko Tsukamoto, Yuka Kadomatsu, Yoko Kubo, Rieko Okada, Mako Nagayoshi, Takashi Tamura, Asahi Hishida, Masahiro Nakatochi, Tae Sasakabe, Shuji Hashimoto, Hidetaka Eguchi, Yukihide Momozawa, Hiroaki Ikezaki, Masayuki Murata, Norihiro Furusyo, Keitaro Tanaka, Megumi Hara, Yuichiro Nishida, Keitaro Matsuo, Hidemi Ito, Isao Oze, Haruo Mikami, Yohko Nakamura, Miho Kusakabe, Toshiro Takezaki, Rie Ibusuki, Ippei Shimoshikiryo, Sadao Suzuki, Takeshi Nishiyama, Miki Watanabe, Teruhide Koyama, Etsuko Ozaki, Isao Watanabe, Kiyonori Kuriki, Yoshikuni Kita, Hirotsugu Ueshima, Kenji Matsui, Kokichi Arisawa, Hirokazu Uemura, Sakurako Katsuura-Kamano, Sho Nakamura, Hiroto Narimatsu, Nobuyuki Hamajima, Hideo Tanaka, Kenji Wakai

**Affiliations:** 1 Department of Preventive Medicine, Nagoya University Graduate School of Medicine, Nagoya, Japan; 2 Department of Oral Epidemiology, Hiroshima University Graduate School of Biomedical and Health Sciences, Hiroshima, Japan; 3 Department of Public Health, Aichi Medical University School of Medicine, Aichi, Japan; 4 Public Health Informatics Unit, Department of Integrated Health Sciences, Nagoya University Graduate School of Medicine, Nagoya, Japan; 5 Department of Hygiene, Fujita Health University School of Medicine, Aichi, Japan; 6 Diagnosis and Therapeutics of Intractable Diseases and Intractable Disease Research Center, Juntendo University Graduate School of Medicine, Tokyo, Japan; 7 Laboratory for Genotyping Development, RIKEN Center for Integrative Medical Sciences, Yokohama, Japan; 8 Department of Comprehensive General Internal Medicine, Kyushu University Graduate School, Faculty of Medical Sciences, Fukuoka, Japan; 9 Department of General Internal Medicine, Kyushu University Hospital, Fukuoka, Japan; 10 Department of Environmental Medicine and Infectious Diseases, Kyushu University Graduate School of Medical Sciences, Fukuoka, Japan; 11 Department of Preventive Medicine, Faculty of Medicine, Saga University, Saga, Japan; 12 Division of Cancer Epidemiology and Prevention, Aichi Cancer Center Research Institute, Nagoya, Japan; 13 Department of Cancer Epidemiology, Nagoya University Graduate School of Medicine, Nagoya, Japan; 14 Division of Cancer Information and Control, Aichi Cancer Center Research Institute, Nagoya, Japan; 15 Department of Descriptive Cancer Epidemiology, Nagoya University Graduate School of Medicine, Nagoya, Japan; 16 Cancer Prevention Center, Chiba Cancer Center Research Institute, Chiba, Japan; 17 Department of International Island and Community Medicine, Kagoshima University Graduate School of Medical and Dental Sciences, Kagoshima, Japan; 18 Department of Public Health, Nagoya City University Graduate School of Medical Sciences, Nagoya, Japan; 19 Department of Epidemiology for Community Health and Medicine, Kyoto Prefectural University of Medicine, Kyoto, Japan; 20 Laboratory of Public Health, Division of Nutritional Sciences, School of Food and Nutritional Sciences, University of Shizuoka, Shizuoka, Japan; 21 Faculty of Nursing Science, Tsuruga Nursing University, Fukui, Japan; 22 Center for Epidemiologic Research in Asia, Shiga University of Medical Science, Otsu, Japan; 23 Division of Bioethics and Healthcare Law, The National Cancer Center Japan, Tokyo, Japan; 24 Department of Preventive Medicine, Tokushima University Graduate School of Biomedical Sciences, Tokushima, Japan; 25 College of Nursing Art and Science, University of Hyogo, Akashi, Japan; 26 Cancer Prevention and Control Division, Kanagawa Cancer Center Research Institute, Kanagawa, Japan; 27 School of Health of Innovation, Kanagawa University of Human Services, Kanagawa, Japan; 28 Department of Healthcare Administration, Nagoya University Graduate School of Medicine, Nagoya, Japan; 29 Shiga University of Medical Science, Otsu, Japan

**Keywords:** study profile, cohort study, gene–environment interactions, cancer, J-MICC

## Abstract

**Background:**

The Japan Multi-institutional Collaborative Cohort (J-MICC) study was launched in 2005 to examine gene–environment interactions in lifestyle-related diseases, including cancers, among the Japanese. This report describes the study design and baseline profile of the study participants.

**Methods:**

The participants of the J-MICC Study were individuals aged 35 to 69 years enrolled from respondents to study announcements in specified regions, inhabitants attending health checkup examinations provided by local governments, visitors at health checkup centers, and first-visit patients at a cancer hospital in Japan. At the time of the baseline survey, from 2005 to 2014, we obtained comprehensive information regarding demographics, education, alcohol consumption, smoking, sleeping, exercise, food intake frequency, medication and supplement use, personal and family disease history, psychological stress, and female reproductive history and collected peripheral blood samples.

**Results:**

The baseline survey included 92,610 adults (mean age: 55.2 [standard deviation, 9.4] years, 44.1% men) from 14 study regions in 12 prefectures. The participation rate was 33.5%, with participation ranging from 19.7% to 69.8% in different study regions. The largest number of participants was in the age groups of 65–69 years for men and 60–64 years for women. There were differences in body mass index, educational attainment, alcohol consumption, smoking, and sleep duration between men and women.

**Conclusions:**

The J-MICC Study collected lifestyle and clinical data and biospecimens from over 90,000 participants. This cohort is expected to be a valuable resource for the national and international scientific community in providing evidence to support longer healthy lives.

## INTRODUCTION

Since 1981, cancer, a lifestyle-related disease, has been the leading cause of death in Japan and has continued to be a substantial public health burden. In Japan, several cohort studies were started in the 1980s and 1990s to identify factors contributing to the decrease in the incidence of lifestyle-related diseases represented by cancer. Representative large-scale cohort studies for cancer prevention include the following. From 1988 through 1990, the Japan Collaborative Cohort (JACC) study was established, covering 45 regions in Japan, and has followed up approximately 110,000 individuals aged 40 to 79 years.^1^ From 1990 to 1993, the Japan Public Health Center-based Cohort (JPHC) study began covering 11 public health center regions throughout Japan and has included a total of about 140,000 individuals aged 40 to 69 years.^2^ However, cancer is still the leading cause of death, resulting in approximately 0.37 million deaths (27.4% of all deaths) in 2018.^3^

With recent advances in genotyping techniques, gene–environment interactions in lifestyle-related diseases have begun to be investigated in many epidemiological studies.^4^^–^^8^ This is because most multifactorial diseases are considered to be caused by interactions between hazardous environmental factors and the host genome. Elucidating gene–environment interactions requires long-term cohort studies, which cover the etiologically relevant time period to improve the accuracy of measures of exposures by collecting repeated biologic samples and self-reported information.^9^ Therefore, we launched the Japan Multi-institutional Collaborative Cohort (J-MICC) study in 2005, which includes healthy Japanese individuals. As part of this study, buffy coat, serum, and plasma samples are stored. The J-MICC Study is supported by a research grant for Scientific Research on Special Priority Areas of Cancer from the Japanese Ministry of Education, Culture, Sports, Science and Technology (MEXT).^10^ The aims of this large-scale, population-based, long-term prospective, genome-cohort study are to examine gene–environment interactions in lifestyle-related diseases, especially cancers. While the J-MICC Study Group reported many cross-sectional studies focusing on the gene–environmental interaction for health outcomes, there were no reports of detailed study design and baseline participant age distribution by study region. Therefore, this report describes the study design and profile of the participants at baseline.

## METHODS

### Study design and organization

The J-MICC Study is being conducted under a population-based cohort design, managed by 13 research groups (J-MICC Study Group) from 12 prefectures: Chiba Cancer Center Research Institute, Kanagawa Cancer Center Research Institute, University of Shizuoka, Nagoya City University, Aichi Cancer Center Research Institute, Nagoya University, Tsuruga Nursing University, Shiga University of Medical Science, Kyoto Prefectural University of Medicine, Tokushima University Graduate School, Kyushu University, Saga University, and Kagoshima University. Each group has its own independent research site and conducts a cohort study as part of the J-MICC Study, which allows sites to collect and analyze additional information or samples for their own research purposes. The Steering Committee is organized by a representative from each group to manage and control the progress of the whole J-MICC Study. The chief investigator of the J-MICC Study is the chairperson of the Steering Committee. The central office was established at Nagoya University Graduate School of Medicine, where all J-MICC data and half of all blood samples are preserved. The central office also makes efforts to standardize the processes in each cohort study, supplies common tools (eg, data input system, sample management system, posters, brochures), and maintains the study website (http://www.jmicc.com/). The study protocol of the J-MICC Study was approved by the ethics committee of Nagoya University Graduate School of Medicine (approval number: 253), as well as by each group in the research sites. All participants provided written informed consent after a thorough explanation of the outline and objectives of this study.

### Study participants

**Figure 1.**
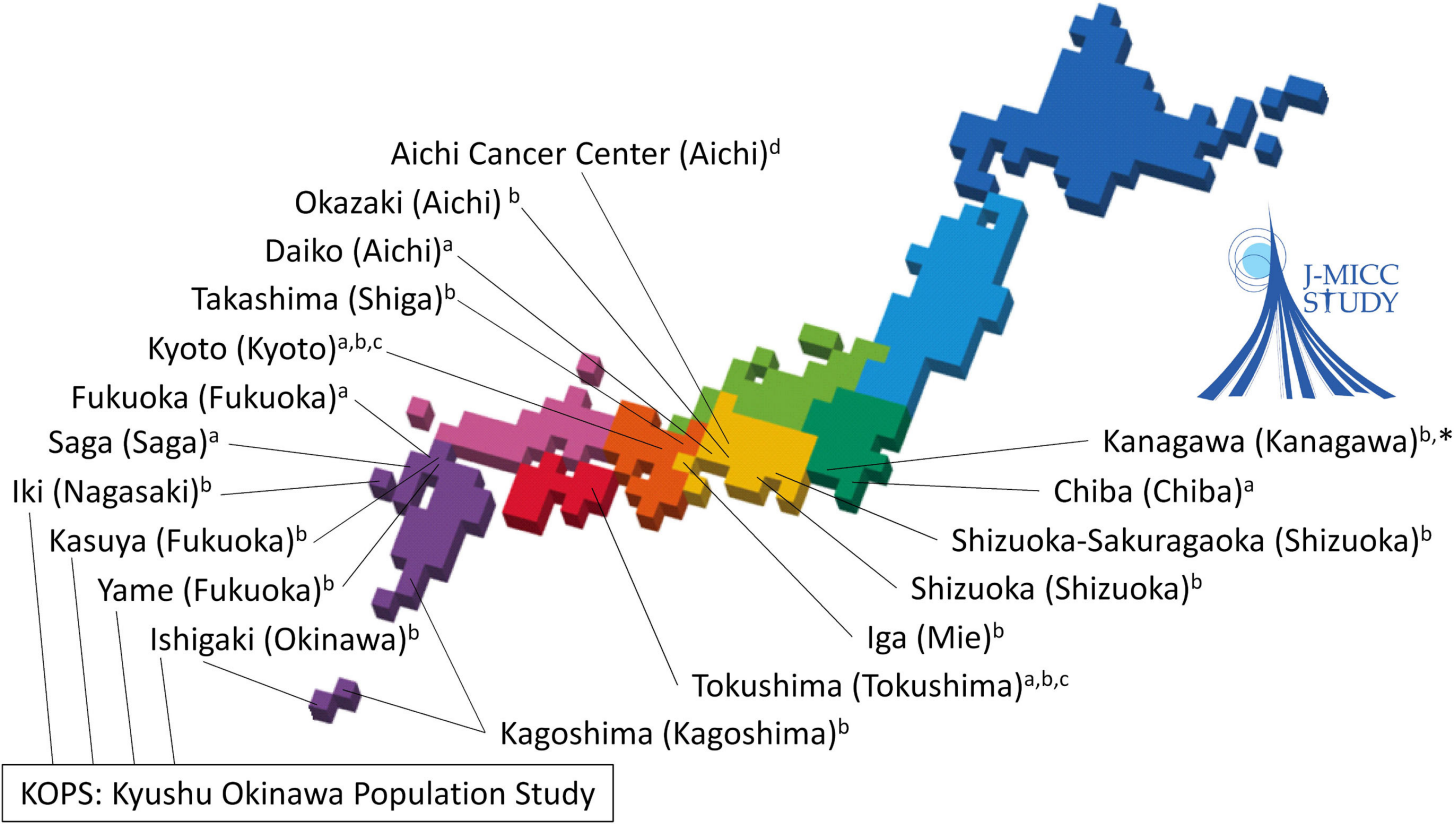
Locations of the study regions of the J-MICC Study. ^*^Baseline survey started in 2016 and ongoing. ^a^Inhabitants in communities; ^b^Health checkup examinees; ^c^Employees of companies or local governments; ^d^First-visit patients at a cancer hospital

The participants of the J-MICC Study were individuals aged 35 through 69 years living in Japan. At the time of the baseline survey, from 2005–2014, the participants completed questionnaires and provided peripheral blood samples. The baseline study participants were recruited from 14 different regions throughout Japan (Figure 1). The study regions included Chiba, Shizuoka-Sakuragaoka, Shizuoka, Okazaki, Aichi Cancer Center, Daiko, Iga, Takashima, Kyoto, Tokushima, Fukuoka, Saga, Kagoshima, and the Kyusyu and Okinawa Population Study (KOPS) area. The subject sources are inhabitants in communities in four regions (Chiba, Daiko, Fukuoka, and Saga), health checkup examinees in seven regions (Shizuoka-Sakuragaoka, Shizuoka, Okazaki, Iga, Takashima, Kagoshima, and KOPS), and first-visit patients at a cancer hospital in Aichi Cancer Center region. In Kyoto and Tokushima regions, the subject sources are inhabitants in communities and health checkup examinees as well as employees of companies or local governments. In six regions (Shizuoka-Sakuragaoka, Okazaki, Takashima, Kyoto, Fukuoka, and KOPS), individuals under 35 and/or over 70 years of age were also recruited as study participants. In the Fukuoka and Saga regions, individuals aged 50 years or older and 40 years or older in principle were recruited, respectively. In the Fukuoka and KOPS regions, the survey started earlier (in 2004) and Kyushu University has been operating in study regions and collaborating with groups in the J-MICC Study. In the Kanagawa region, the baseline survey started in 2016 and is still ongoing.

### Follow-up survey

The secondary survey, which is the same as the baseline survey, is scheduled for approximately 5 years after baseline enrollment, and participants will be followed until 2025. The endpoints of the J-MICC Study are cancer incidence and death from any cause, while incidence of cerebrovascular disease and myocardial infarction has been additionally registered in 10 study regions. As stated,^10^ information regarding endpoints are collected through population-based cancer registries if available, lists of patients at main hospitals in the study regions, mail questionnaires sent to participants, questionnaires at repeated visits to health checkup facilities, notes from death certificates, information from health insurance data, and secondary survey questionnaires. The incidence data other than those from cancer registries are also confirmed using hospital records. When a participant moves out of the study region, the participant’s follow-up is censored.

Longitudinal analyses of the follow-up survey data will be conducted in the near future.

### Data and blood samples collection

At the time of enrollment, the self-administered questionnaire was distributed to the entire target population, which included common questions across all study regions. Table 1 summarizes the common questions covering basic, lifestyle, and clinical characteristics. In addition, blood chemistry data and anthropometric data were obtained from health check-ups at enrollment. The data collected from health check-ups to be sent to the central office were previously reported.^10^

**Table 1.** Summary of common questions in the self-administered questionnaire for all study regions at baseline

Measurements	Measurement lists
Basic characteristics	
Demographics	Sex, age at baseline, height, weight, and weight at the age of 20 years
Education^*^	Educational attainment
Lifestyle characteristics	
Alcohol consumption	Drinking status, age when the individual started drinking, type and amount of alcohol consumed, and drinking frequency
Smoking	Smoking status, age when the individual started smoking, number of cigarettes smoked, and information on passive smoking
Sleeping	Sleeping duration and subjective assessment of sleep
Exercise	Physical activity (including leisure-time, occupational, and household activities)
Diet	Food intake frequency
Clinical characteristics	
Medication and supplements	Types of medications and supplements taken at least once a week
Disease history	Personal and family disease history
Psychological stress	Self-reported stress and stress management
Female reproductive history	Menstruation status, age at the start of menstruation, and information on pregnancy and childbirth

^*^Excluding 3 regions (Iga, Fukuoka, and Kyusyu and Okinawa Population Study regions).

We collected blood samples in a 7-mL vacuum tube for serum and a 7-mL EDTA-2Na-containing vacuum tube for plasma and buffy coat. The blood samples sent to the central office consisted of one tube containing 300 µL of buffy coat, four tubes containing 300 µL of serum, and four tubes containing 300 µL of plasma. Some of the blood specimens will be stored at the central office until the end of the J-MICC Study in 2035.^11^

Baseline data and blood samples (buffy coat, serum, and plasma), anonymized with an identification number (J-MICC ID), are sent to the central office from each participating research group. The secondary survey and follow-up data are linked using the J-MICC ID.

### Statistical analyses

For the baseline profile (excluding the Kanagawa region because of the ongoing baseline survey for 5,000 participants), descriptive statistics were calculated for baseline data regarding sex, age, body mass index (BMI) calculated from self-reported height and weight, educational attainment, alcohol consumption, smoking, sleeping duration, leisure time physical activity, psychological stress, and personal (past and present) disease history. In the case of educational attainment, participants from the Iga, Fukuoka, and KOPS regions were excluded from the analysis because the questionnaire used there did not include this item.

## RESULTS

Among 247,951 eligible individuals, 83,114 (33.5% in total with response rates ranging from 19.7% to 69.8% in different study regions) consented to participate in the baseline survey of the J-MICC Study. Furthermore, by distributing 2,786,327 fliers via mailboxes in four regions, 18,368 respondents were additionally recruited. In total, 101,482 men and women participated in the baseline survey. After excluding 8,294 respondents under 35 or over 70 years of age and 578 who withdrew consent or became ineligible, the remaining 92,610 participants (40,880 men and 51,730 women with an average age of 55.2 [standard deviation, 9.4] years) in the baseline survey were included in the present analysis (the dataset was fixed on February 2, 2020). Among the participants, 90,319 people (97.5% of total participants) consented to the use of their biospecimens in the J-MICC Study, including genomic analysis (90,252 people; 99.9% of total consenters).

**Figure 2.**
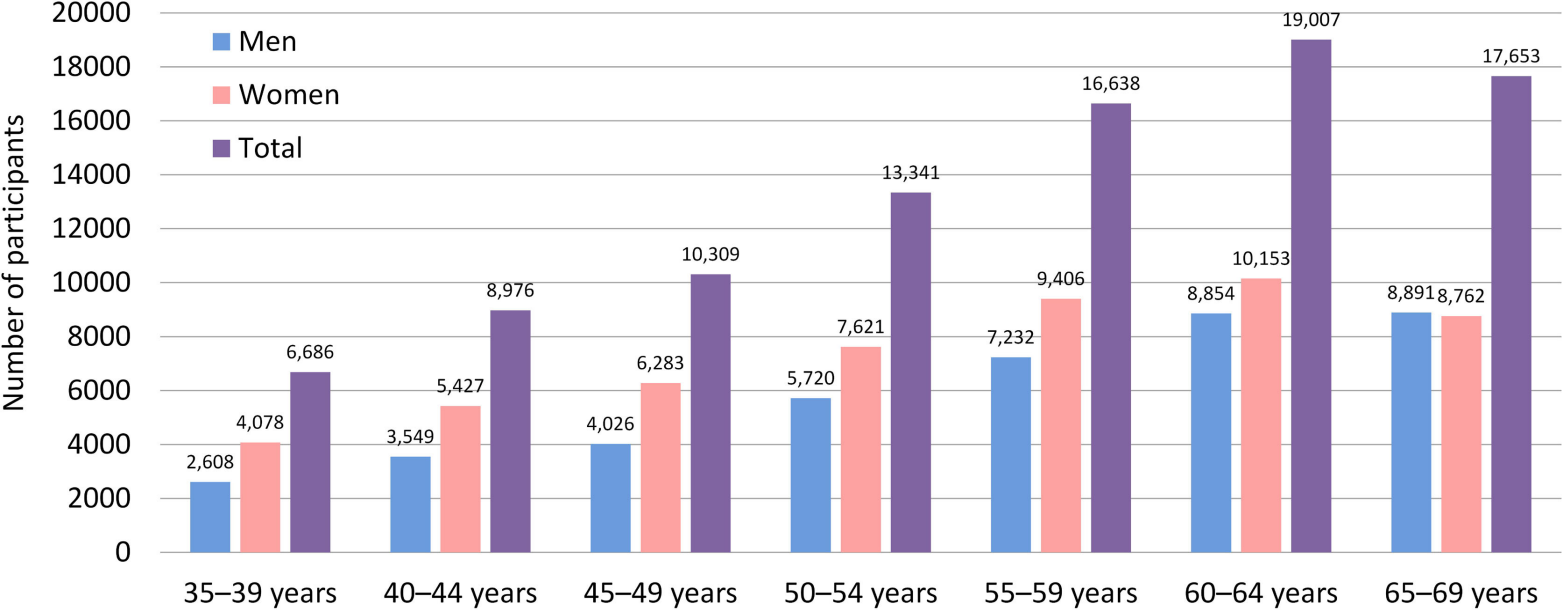
Age-sex distribution of participants in the baseline survey of the J-MICC Study

The age-sex distribution of the participants in the baseline survey is presented in Figure 2. Of the respondents, 7.2% belonged to the 35–39 years age group, 9.7% to the 40–44 years age group, 11.1% to the 45–49 years age group, 14.4% to the 50–54 years age group, 18.0% to the 55–59 years age group, 20.5% to the 60–64 years age group, and 19.1% to the 65–69 years age group. The largest number of participants were in the 65–69 age group for men (21.7% of male participants) and the 60–64 age group for women (19.6% of female participants). The number of female participants was higher than that of male participants for all age categories, except for those aged 65–69.

The age distribution of the male and female participants in the baseline survey by the study regions is shown in Table 2 and Table 3, respectively. The average age of participants who were enrolled in Chiba, Shizuoka-Sakuragaoka, Shizuoka, Okazaki, Aichi Cancer Center, Daiko, Iga, Takashima, Kyoto, Tokushima, Fukuoka, Saga, Kagoshima, and KOPS regions were 53.9, 54.8, 52.2, 55.8, 55.1, 52.5, 51.2, 57.7, 51.6, 50.6, 60.3, 56.0, 57.5, and 54.7 years, respectively. There were considerable differences in age distributions by study region among both men and women. Notably, the percentage of people aged 35 to 39 years ranged from 0.0% to 16.7% in men and from 0.0% to 16.0% in women. Likewise, the percentage of people aged 40 to 44 years and 45 to 49 years ranged from 0.0% to 16.1% and 0.0% to 15.9% in men and from 0.0% to 16.5% and 0.1% to 18.1% in women, respectively.

**Table 2.** Age distribution of male participants in the baseline survey of the J-MICC Study by study region

Study region		Age, years							Total	Average age (SD)
	
		35–39	40–44	45–49	50–54	55–59	60–64	65–69		
Total	*N*	2,608	3,549	4,026	5,720	7,232	8,854	8,891	40,880	56.1
	(%)	(6.4)	(8.7)	(9.8)	(14.0)	(17.7)	(21.7)	(21.7)	(100.0)	(9.3)
Chiba	*N*	293	272	292	347	455	562	697	2,918	55.4
	(%)	(10.0)	(9.3)	(10.0)	(11.9)	(15.6)	(19.3)	(23.9)	(100.0)	(10.1)
Shizuoka-Sakuragaoka	*N*	240	277	286	435	521	808	563	3,130	55.6
	(%)	(7.7)	(8.8)	(9.1)	(13.9)	(16.6)	(25.8)	(18.0)	(100.0)	(9.3)
Shizuoka	*N*	265	437	542	585	782	483	312	3,406	52.7
	(%)	(7.8)	(12.8)	(15.9)	(17.2)	(23.0)	(14.2)	(9.2)	(100.0)	(8.7)
Okazaki	*N*	262	271	353	376	449	824	955	3,490	56.7
	(%)	(7.5)	(7.8)	(10.1)	(10.8)	(12.9)	(23.6)	(27.4)	(100.0)	(9.7)
Aichi Cancer Center	*N*	192	225	301	462	837	1,119	1,159	4,295	58.2
	(%)	(4.5)	(5.2)	(7.0)	(10.8)	(19.5)	(26.1)	(27.0)	(100.0)	(8.6)
Daiko	*N*	190	161	185	195	206	241	284	1,462	53.6
	(%)	(13.0)	(11.0)	(12.7)	(13.3)	(14.1)	(16.5)	(19.4)	(100.0)	(10.3)
Iga	*N*	100	94	76	115	89	94	68	636	51.3
	(%)	(15.7)	(14.8)	(11.9)	(18.1)	(14.0)	(14.8)	(10.7)	(100.0)	(9.8)
Takashima	*N*	72	58	78	87	161	305	477	1,238	59.3
	(%)	(5.8)	(4.7)	(6.3)	(7.0)	(13.0)	(24.6)	(38.5)	(100.0)	(9.1)
Kyoto	*N*	431	344	360	369	346	385	341	2,576	51.7
	(%)	(16.7)	(13.4)	(14.0)	(14.3)	(13.4)	(14.9)	(13.2)	(100.0)	(10.3)
Tokushima	*N*	224	228	191	225	218	221	107	1,414	50.8
	(%)	(15.8)	(16.1)	(13.5)	(15.9)	(15.4)	(15.6)	(7.6)	(100.0)	(9.6)
Fukuoka	*N*	0	0	0	782	1,131	1,256	1,287	4,456	60.5
	(%)	(0.0)	(0.0)	(0.0)	(17.5)	(25.4)	(28.2)	(28.9)	(100.0)	(5.4)
Saga	*N*	0	569	591	793	1,048	1,025	1,052	5,078	56.5
	(%)	(0.0)	(11.2)	(11.6)	(15.6)	(20.6)	(20.2)	(20.7)	(100.0)	(8.2)
Kagoshima	*N*	79	230	366	435	465	738	847	3,160	57.5
	(%)	(2.5)	(7.3)	(11.6)	(13.8)	(14.7)	(23.4)	(26.8)	(100.0)	(8.6)
KOPS	*N*	260	383	405	514	524	793	742	3,621	55.3
	(%)	(7.2)	(10.6)	(11.2)	(14.2)	(14.5)	(21.9)	(20.5)	(100.0)	(9.6)

KOPS, Kyusyu and Okinawa Population Study; SD, standard deviation.

**Table 3.** Age distribution of female participants in the baseline survey of the J-MICC Study by study region

Study region		Age, years							Total	Average age (SD)
	
		35–39	40–44	45–49	50–54	55–59	60–64	65–69		
Total	*N*	4,078	5,427	6,283	7,621	9,406	10,153	8,762	51,730	54.6
	(%)	(7.9)	(10.5)	(12.1)	(14.7)	(18.2)	(19.6)	(16.9)	(100.0)	(9.4)
Chiba	*N*	636	607	703	732	854	894	750	5,176	53.1
	(%)	(12.3)	(11.7)	(13.6)	(14.1)	(16.5)	(17.3)	(14.5)	(100.0)	(9.8)
Shizuoka-Sakuragaoka	*N*	215	288	339	343	406	456	340	2,387	53.6
	(%)	(9.0)	(12.1)	(14.2)	(14.4)	(17.0)	(19.1)	(14.2)	(100.0)	(9.5)
Shizuoka	*N*	195	198	290	296	331	186	104	1,600	51.2
	(%)	(12.2)	(12.4)	(18.1)	(18.5)	(20.7)	(11.6)	(6.5)	(100.0)	(8.6)
Okazaki	*N*	220	331	411	411	506	657	524	3,060	54.7
	(%)	(7.2)	(10.8)	(13.4)	(13.4)	(16.5)	(21.5)	(17.1)	(100.0)	(9.4)
Aichi Cancer Center	*N*	572	656	759	659	791	807	562	4,806	52.3
	(%)	(11.9)	(13.6)	(15.8)	(13.7)	(16.5)	(16.8)	(11.7)	(100.0)	(9.6)
Daiko	*N*	570	489	542	472	470	558	589	3,690	52.1
	(%)	(15.4)	(13.3)	(14.7)	(12.8)	(12.7)	(15.1)	(16.0)	(100.0)	(10.3)
Iga	*N*	97	121	127	149	137	104	51	786	51.0
	(%)	(12.3)	(15.4)	(16.2)	(19.0)	(17.4)	(13.2)	(6.5)	(100.0)	(8.7)
Takashima	*N*	221	139	175	232	347	575	601	2,290	56.8
	(%)	(9.7)	(6.1)	(7.6)	(10.1)	(15.2)	(25.1)	(26.2)	(100.0)	(9.8)
Kyoto	*N*	544	552	588	488	444	576	428	3,620	51.5
	(%)	(15.0)	(15.2)	(16.2)	(13.5)	(12.3)	(15.9)	(11.8)	(100.0)	(10.0)
Tokushima	*N*	164	169	161	166	148	136	82	1,026	50.4
	(%)	(16.0)	(16.5)	(15.7)	(16.2)	(14.4)	(13.3)	(8.0)	(100.0)	(9.5)
Fukuoka	*N*	0	0	3	1,051	1,617	1,538	1,428	5,637	60.0
	(%)	(0.0)	(0.0)	(0.1)	(18.6)	(28.7)	(27.3)	(25.3)	(100.0)	(5.3)
Saga	*N*	0	921	940	1,150	1,409	1,374	1,196	6,990	55.6
	(%)	(0.0)	(13.2)	(13.4)	(16.5)	(20.2)	(19.7)	(17.1)	(100.0)	(8.2)
Kagoshima	*N*	60	313	497	617	850	1,074	1,067	4,478	57.5
	(%)	(1.3)	(7.0)	(11.1)	(13.8)	(19.0)	(24.0)	(23.8)	(100.0)	(8.1)
KOPS	*N*	584	643	748	855	1,096	1,218	1,040	6,184	54.3
	(%)	(9.4)	(10.4)	(12.1)	(13.8)	(17.7)	(19.7)	(16.8)	(100.0)	(9.7)

KOPS, Kyusyu and Okinawa Population Study; SD, standard deviation.

Basic, lifestyle, and clinical characteristics of the male and female participants in the baseline survey by age category are summarized in Table 4 and Table 5, respectively. There were differences in BMI, educational attainment, alcohol consumption, smoking, and sleep duration between men and women. The percentage of overweight adults (BMI 25.0–29.9 kg/m^2^) was 1.7 times higher for men (26.7%) than for women (15.8%). Approximately 40% of men had ≥16 years of education compared with 13.5% of women. The likelihood of being a former drinker and sleeping ≥9 hours/day increased gradually with higher age categories among men, but no such association was observed among women. The likelihood of being a never smoker increased gradually with higher age categories among women, but no such association was observed among men. For leisure time physical activity, psychological stress, and history of disease, the percentages of participants with ≥300 min/week of leisure-time activity, those perceiving no stress, and those with a history of diseases increased gradually with higher age categories among both men and women. For cancer, when patients first seen at the Aichi Cancer Center, a cancer hospital, were excluded from the analysis, cancer prevalence fell from 8.5% to 4.5% for men and from 7.1% to 5.1% for women.

**Table 4.** Baseline characteristics of male participants of the J-MICC Study according to age category (*N* = 40,880)

Characteristic	Age, years														Total	

	35–39		40–44		45–49		50–54		55–59		60–64		65–69			

	*N*	%	*N*	%	*N*	%	*N*	%	*N*	%	*N*	%	*N*	%	*N*	%
Body mass index, kg/m^2^																
<18.5	87	3.3	99	2.8	105	2.6	136	2.4	207	2.9	281	3.2	298	3.4	1,213	3.0
18.5–24.9	1,742	66.8	2,288	64.5	2,553	63.4	3,618	63.3	4,840	66.9	6,080	68.7	6,291	70.8	27,412	67.1
25.0–29.9	640	24.6	952	26.9	1,198	29.8	1,730	30.3	1,989	27.5	2,278	25.7	2,141	24.1	10,928	26.7
≥30.0	137	5.3	206	5.8	169	4.2	234	4.1	194	2.7	209	2.4	152	1.7	1,301	3.2
Educational attainment, years																
<10	45	2.0	94	3.1	100	2.8	197	4.6	460	8.4	944	14.2	1,482	22.0	3,322	10.4
10–15	1,005	44.8	1,442	47.1	1,674	47.4	2,052	47.8	2,843	52.1	3,503	52.6	3,324	49.4	15,843	49.5
≥16	1,193	53.2	1,526	49.8	1,758	49.8	2,048	47.7	2,156	39.5	2,213	33.2	1,927	28.6	12,821	40.1
Alcohol consumption																
Current drinkers	1,902	72.9	2,679	75.5	3,163	78.6	4,465	78.2	5,500	76.1	6,646	75.1	6,507	73.3	30,862	75.6
Former drinkers	36	1.4	74	2.1	87	2.2	177	3.1	281	3.9	423	4.8	552	6.2	1,630	4.0
Never drinkers	670	25.7	794	22.4	775	19.3	1,070	18.7	1,443	20.0	1,781	20.1	1,821	20.5	8,354	20.5
Smoking																
Current smokers	879	34.0	1,310	37.1	1,416	35.3	2,052	36.0	2,364	32.7	2,368	26.8	1,772	20.0	12,161	29.8
Former smokers	713	27.6	1,066	30.2	1,393	34.7	2,206	38.7	3,097	42.9	4,160	47.1	4,382	49.4	17,017	41.7
Never smokers	990	38.3	1,159	32.8	1,200	29.9	1,444	25.3	1,763	24.4	2,305	26.1	2,722	30.7	11,583	28.4
Sleep duration, hour/day																
<4.0	10	0.4	12	0.3	9	0.2	8	0.1	12	0.2	11	0.1	18	0.2	80	0.2
4.0–4.9	77	3.0	74	2.1	73	1.8	71	1.2	70	1.0	113	1.3	85	1.0	563	1.4
5.0–5.9	364	14.0	452	12.7	469	11.7	639	11.2	618	8.6	555	6.3	560	6.3	3,657	9.0
6.0–6.9	1,027	39.5	1,400	39.5	1,553	38.6	1,999	35.0	2,415	33.4	2,372	26.8	2,087	23.5	12,853	31.5
7.0–7.9	801	30.8	1,133	32.0	1,374	34.2	2,105	36.8	2,710	37.5	3,249	36.7	3,117	35.1	14,489	35.5
8.0–8.9	292	11.2	437	12.3	485	12.1	797	14.0	1,230	17.0	2,167	24.5	2,443	27.5	7,851	19.2
≥9.0	30	1.2	38	1.1	59	1.5	94	1.6	168	2.3	380	4.3	572	6.4	1,341	3.3
Leisure time physical activity, min/week																
<30	1,078	42.7	1,544	44.6	1,631	41.7	2,206	39.6	2,555	36.4	2,386	27.8	1,878	22.1	13,278	33.6
30–59	231	9.2	302	8.7	303	7.7	405	7.3	487	6.9	452	5.3	305	3.6	2,485	6.3
60–119	449	17.8	550	15.9	696	17.8	964	17.3	1,203	17.1	1,206	14.1	951	11.2	6,019	15.2
120–179	255	10.1	361	10.4	406	10.4	626	11.2	828	11.8	937	10.9	906	10.7	4,319	10.9
180–299	206	8.2	280	8.1	354	9.0	560	10.0	780	11.1	1,100	12.8	1,091	12.9	4,371	11.1
≥300	304	12.0	427	12.3	525	13.4	815	14.6	1,168	16.6	2,496	29.1	3,349	39.5	9,084	23.0
Psychological stress during the last year																
No stress	30	1.2	70	2.0	108	2.8	180	3.2	331	4.7	680	7.8	955	10.9	2,354	5.9
Low stress	375	15.0	494	14.3	627	16.0	1,086	19.4	1,654	23.3	3,011	34.5	3,475	39.6	10,722	26.8
Moderate stress	1,254	50.2	1,674	48.6	1,936	49.3	2,713	48.5	3,443	48.5	3,800	43.6	3,510	40.0	18,330	45.8
High stress	838	33.6	1,207	35.0	1,253	31.9	1,612	28.8	1,676	23.6	1,226	14.1	840	9.6	8,652	21.6
Personal disease history																
Diabetes	28	1.1	106	3.0	185	4.6	352	6.6	744	11.1	1,162	14.0	1,194	14.4	3,771	9.7
Hypertension	105	4.0	264	7.4	559	13.9	1,087	20.2	1,835	27.0	2,847	34.1	3,305	39.1	10,002	25.6
Dyslipidemia	147	5.6	352	9.9	596	14.9	923	17.2	1,320	19.7	1,618	19.7	1,569	19.0	6,525	16.9
Coronary heart disease	11	0.4	25	0.7	57	1.4	120	2.3	249	3.8	472	5.8	662	8.1	1,596	4.2
Stroke	4	0.2	16	0.5	26	0.6	78	1.5	150	2.3	297	3.6	365	4.5	936	2.4
Cancer	52	2.1	76	2.2	132	3.4	257	5.1	577	9.0	909	11.4	1,159	14.7	3,162	8.5

**Table 5.** Baseline characteristics of female participants of the J-MICC Study according to age category (*N* = 51,730)

Characteristic	Age, years														Total	

	35–39		40–44		45–49		50–54		55–59		60–64		65–69			

	*N*	%	*N*	%	*N*	%	*N*	%	*N*	%	*N*	%	*N*	%	*N*	%
Body mass index, kg/m^2^																
<18.5	746	18.4	706	13.0	639	10.2	652	8.6	766	8.2	725	7.2	562	6.4	4,796	9.3
18.5–24.9	2,900	71.4	3,935	72.6	4,582	73.0	5,581	73.3	6,772	72.1	7,377	72.8	6,154	70.4	37,301	72.2
25.0–29.9	323	8.0	630	11.6	889	14.2	1,175	15.4	1,614	17.2	1,794	17.7	1,735	19.8	8,160	15.8
≥30.0	90	2.2	147	2.7	166	2.6	208	2.7	242	2.6	243	2.4	294	3.4	1,390	2.7
Educational attainment, years																
<10	58	1.7	60	1.3	91	1.7	190	3.4	542	8.3	1,052	14.5	1,523	24.6	3,516	9.0
10–15	2,413	71.4	3,682	79.2	4,367	81.0	4,471	80.6	5,273	81.0	5,619	77.5	4,314	69.8	30,139	77.5
≥16	908	26.9	905	19.5	931	17.3	887	16.0	696	10.7	582	8.0	346	5.6	5,255	13.5
Alcohol consumption																
Current drinkers	1,959	48.1	2,607	48.1	2,896	46.2	3,077	40.4	3,219	34.3	3,137	31.0	2,276	26.0	19,171	37.1
Former drinkers	158	3.9	124	2.3	101	1.6	150	2.0	173	1.8	176	1.7	157	1.8	1,039	2.0
Never drinkers	1,956	48.0	2,690	49.6	3,275	52.2	4,386	57.6	5,999	63.9	6,817	67.3	6,320	72.2	31,443	60.9
Smoking																
Current smokers	442	10.9	557	10.3	649	10.4	697	9.2	653	7.0	474	4.7	252	2.9	3,724	7.2
Former smokers	567	14.0	544	10.1	610	9.8	649	8.6	613	6.6	514	5.1	337	3.9	3,834	7.5
Never smokers	3,042	75.1	4,292	79.6	4,975	79.8	6,230	82.2	8,089	86.5	9,124	90.2	8,145	93.3	43,897	85.3
Sleep duration, hour/day																
<4.0	7	0.2	17	0.3	21	0.3	22	0.3	15	0.2	28	0.3	33	0.4	143	0.3
4.0–4.9	79	1.9	127	2.3	158	2.5	154	2.0	181	1.9	171	1.7	153	1.7	1,023	2.0
5.0–5.9	474	11.6	853	15.7	1,093	17.4	1,226	16.1	1,211	12.9	1,022	10.1	958	10.9	6,837	13.2
6.0–6.9	1,451	35.6	2,201	40.6	2,722	43.4	3,242	42.6	3,540	37.7	3,281	32.3	2,572	29.4	19,009	36.8
7.0–7.9	1,348	33.1	1,624	30.0	1,776	28.3	2,293	30.1	3,302	35.2	3,886	38.3	3,241	37.0	17,470	33.8
8.0–8.9	630	15.5	546	10.1	461	7.3	629	8.3	1,060	11.3	1,592	15.7	1,576	18.0	6,494	12.6
≥9.0	86	2.1	53	1.0	45	0.7	49	0.6	83	0.9	166	1.6	223	2.5	705	1.4
Leisure time physical activity, min/week																
<30	1,931	49.2	2,602	49.2	2,811	46.3	3,071	41.7	3,235	35.7	2,795	28.9	2,123	25.8	18,568	37.4
30–59	346	8.8	442	8.4	465	7.7	510	6.9	574	6.3	486	5.0	408	5.0	3,231	6.5
60–119	508	12.9	733	13.9	763	12.6	1,020	13.9	1,190	13.1	1,193	12.3	970	11.8	6,377	12.9
120–179	384	9.8	566	10.7	692	11.4	856	11.6	1,150	12.7	1,312	13.6	1,059	12.9	6,019	12.1
180–299	311	7.9	423	8.0	586	9.7	825	11.2	1,129	12.5	1,275	13.2	1,163	14.1	5,712	11.5
≥300	446	11.4	522	9.9	751	12.4	1,082	14.7	1,777	19.6	2,600	26.9	2,499	30.4	9,677	19.5
Psychological stress during the last year																
No stress	23	0.6	35	0.7	61	1.0	110	1.5	180	1.9	358	3.6	480	5.5	1,247	2.5
Low stress	435	11.0	538	10.2	708	11.5	1,023	13.7	1,559	16.9	2,088	20.9	2,191	25.3	8,542	16.8
Moderate stress	1,897	47.8	2,553	48.3	2,968	48.4	3,676	49.4	4,623	50.0	4,971	49.7	4,235	48.8	24,923	49.1
High stress	1,615	40.7	2,163	40.9	2,398	39.1	2,632	35.4	2,879	31.2	2,578	25.8	1,764	20.3	16,029	31.6
Personal disease history																
Diabetes	18	0.4	39	0.7	73	1.2	156	2.2	375	4.3	538	5.7	555	6.9	1,754	3.6
Hypertension	61	1.5	155	2.9	359	5.7	829	11.5	1,659	18.9	2,445	25.6	2,618	32.0	8,126	16.4
Dyslipidemia	79	1.9	186	3.4	347	5.5	817	11.3	1,642	18.7	2,410	25.4	2,349	28.8	7,830	15.9
Coronary heart disease	8	0.2	12	0.2	28	0.5	67	1.0	146	1.7	334	3.6	359	4.5	954	2.0
Stroke	12	0.3	14	0.3	34	0.5	58	0.8	106	1.2	192	2.1	211	2.6	627	1.3
Cancer	144	3.7	218	4.3	327	5.5	447	6.6	657	7.9	845	9.3	685	8.8	3,323	7.1

## DISCUSSION

The present report describes the study design and profile of participants in the baseline survey of the J-MICC Study, which incorporates 14 study regions from 12 prefectures. We found notable differences in the age distributions of the participants among the study region. There are two possible reasons. First, in the Fukuoka and Saga regions, the participants originally enrolled in the J-MICC Study were in principle limited to adults aged 50 years or older and 40 years or older, respectively. Second, according to the 2010 (the middle year of the baseline survey period) National Census of Japan, there was a 1.6-fold difference in the percentage of the population aged 65 years and older between the lowest (17.4%) and highest (27.0%) in the 12 prefectures including the study regions of the J-MICC Study.

Based on the obtained informed consent, we have already analyzed genomic information from a total of 14,539 participants who were selected to be genotyped from 13 study regions (except for the Iga region). Up to March 2020, we have already published several papers regarding genome-wide association analysis or gene–environmental interaction for health outcomes.^12^^–^^22^ Moreover, as a bioresources support system, we provide support for studies using biospecimens and data that were collected at the baseline survey of the J-MICC Study, which was also included in the informed consent. Our support includes providing biological resources and data regarding 92,000 individuals and genotype data for genome-wide association study from approximately 14,000 individuals. Information on support content is provided on the Platform of Supporting Cohort Study and Biospecimen Analysis webpage (http://cohort.umin.jp/english/about/bio-resource.html).

The main strengths of the J-MICC Study are as follows. First, we comprehensively collected data on both living circumstances and genomic information as risk factors of cancer and other lifestyle-related diseases. Such information would be useful to establish personalized or tailor-made lifestyle-related disease prevention methods. Our data might also be useful as a reference tool, because it allows to gain access to data on genotype distributions in a large, healthy Japanese population. Second, the J-MICC Study (started in 2005) was the first Japanese genome-cohort study collecting data from all over Japan, mainly in the western regions. The Japan Public Health Center-based Prospective Study for the Next Generation (JPHC-NEXT), a genome-cohort of over 100,000 people, was launched 6 years later.^23^ This large-scale, population-based prospective study has been designed to identify risk factors for lifestyle-related diseases, which can in turn contribute to the extension of healthy life expectancy and personalized healthcare. The J-MICC and JPHC-NEXT have conducted a validity study by examining all questionnaires for integrated analysis. Additionally, the Tohoku Medical Megabank Community-Based Cohort Study (TMM CommCohort Study) that began in 2013 was a large scale population-based prospective genome cohort mainly in the east coast of Miyagi and Iwate Prefectures in the Tohoku region to assess the long term impact of the Great East Japan Earthquake and to establish personalized prevention based on the genome, metabolome, and other omics information.^24^ The JPHC-NEXT, TMM CommCohort Study, Tsuruoka Metabolomics Cohort Study,^25^ and Yamagata Molecular Epidemiological Cohort Study^26^ use the same or similar questionnaire established in the J-MICC Study. In the near future, the integration of the J-MICC, JPHC-NEXT, TMM CommCohort Study, and others will finally set up a 300,000-strong cohort research base representing the whole of Japan.

Some potential limitations of the J-MICC Study should also be noted. First, the participation rate was not particularly high (33.5%); hence, our results may have been affected by selection bias. One possible explanation could be that the research sites targeting community inhabitants only mailed invitation letters or distributed leaflets for recruitment. Even in the research sites targeting health checkup examinees, many sites recruited participants only by sending a direct request for survey participation along with the health checkup invitation letter. Second, there were differences between research sites in terms of the recruitment methods (eg, mailing invitation letters or distributing leaflets to the general populations). Also, in some of the research sites, the participants received incentives, such as a small honorarium, which can link to selection bias. These differences, in turn, might be the reason why the region-specific participation rate ranged from 19.7% to 69.8%. Third, it is possible that health check-up attendees are more health conscious, suggesting a healthy volunteer effect in this cohort. For example, current smoking rates among this cohort (male 29.8%, female 7.2%) were slightly lower than those in the 2010 National Health and Nutrition Survey (NHNS) in Japan (male 32.2%, female 8.4%). Furthermore, with respect to history of disease, the rates of diabetes and hypertension among this cohort (diabetes: male 9.7%, female 3.6%; hypertension: male 25.6%, female 16.4%) were also lower compared to those in the 2010 NHNS in Japan (diabetes: male 16.6%, female 9.2%; hypertension: male 57.6%, female 42.2%). Thus, any generalizability of the study findings should be considered with caution. Fourth, our study did not include people over 70 years old. Our study population was 35–69 years of age, and at the beginning of the study, this age group had a greater weight of cancer incidence. Caution should be exercised while extending our findings to the general adult population, including the older adults. Lastly, the incidence rate of early-onset cancers in data from the Aichi Cancer Center will likely be higher than that in other study regions because first-visit patients were recruited at this cancer hospital. The Kanagawa region started the baseline survey about 10 years later than the region where the survey was first launched, so the impact of historical background of this region on the participant characteristics may be different. Thus, there was a problem in simply combining the data from all study regions in the analyses; hence, stratified and sensitivity analyses should be performed in future analyses.

In conclusion, in the J-MICC Study, lifestyle and clinical data and biospecimens were collected from more than 90,000 participants. The present report indicated the study design and identified the baseline characteristics of participants of the J-MICC study. This cohort is expected to be a valuable resource for the national and international scientific community in providing evidence supporting longer healthy lives.
